# Multiport epidural catheter without port and incomplete marking

**DOI:** 10.4103/0019-5049.68387

**Published:** 2010

**Authors:** Suman Lata Gupta, Sandeep Kumar Mishra, Lenin Babu Elakkumanan, Krishnappa Sudeep

**Affiliations:** Department of Anaesthesiology & Critical Care, Jawaharlal Institute of Post-Graduate Medical Education and Research, Pondicherry, India

**Figure 1:**
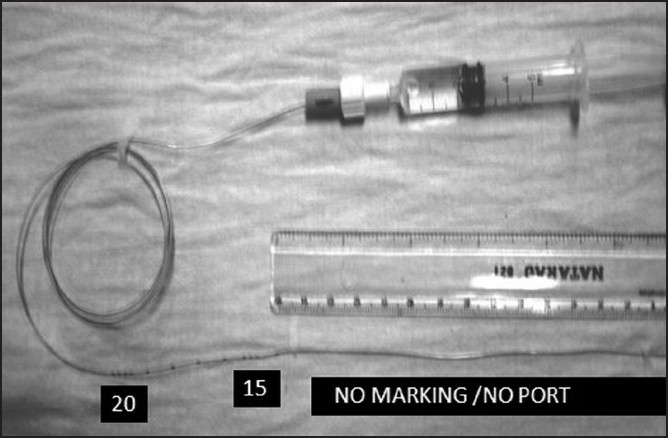
Defective epidural catheter

Sir,

We would like to report a faulty epidural catheter responsible for failure to inject local anaesthestics through it. A case of 50-year-old woman in advanced carcinoma of the cervix posted for Wertheim’s hysterectomy, planned under combined epidural-general anaesthesia. Monitors were connected after securing an intravenous access. Localisation of the epidural space was achieved via the L2-L3 interspace with the patient in the lateral position. A perifix continuous Anaesthesia kit was used. An 18-gauge Tuohy epidural needle was placed in the epidural space without difficulty using the loss-of resistance technique of air. Epidural catheter was inserted through the epidural needle. After inserting few centimeters, it was noticed that markings of the epidural catheter were absent up to 14 cm. (just after three marking i.e., at 15 cm). Catheter is inserted up to 15 cm marking with approximately 4 cm inside epidural space. Injection of the test dose via the catheter was impossible. Incremental withdrawal of the catheter did not correct this situation. The epidural catheter was eventually withdrawn completely. It was not possible with subsequent attempts to flush the catheter. Close inspection of the epidural catheter assembly unit showed that a complete absence of multiple port at the distal end (helical “eyes”) with incomplete markings [Figure 1]. Pre-insertion checking of the catheter and flushing the catheter (“injection test) would have averted this incident. Difficult or impossible injection via the epidural catheter can be a result commonly from mechanical obstruction of the epidural catheter at various levels, like accidental kinking, knotting and malposition of the catheter, occasional manufacturing defects of the catheter[1,2] (defect of the screw-cap connector) can lead to this problem. Before inserting catheter, “injection test” should be performed whenever feasible, not only for epidural catheter but also for other catheter like central venous catheter.
